# Orientation-Dependent Reflection of Structurally Coloured Butterflies

**DOI:** 10.3390/biomimetics5010005

**Published:** 2020-02-03

**Authors:** Sigrid Zobl, Bodo D. Wilts, Willi Salvenmoser, Peter Pölt, Ille C. Gebeshuber, Thorsten Schwerte

**Affiliations:** 1 Institute of Zoology, University of Innsbruck, 6020 Innsbruck, Austria; willi.salvenmoser@uibk.ac.at (W.S.); Thorsten.Schwerte@uibk.ac.at (T.S.); 2 Adolphe Merkle Institute, University of Fribourg, 1700 Fribourg, Switzerland; bodo.wilts@unifr.ch; 3 Center for Molecular Biosciences Innsbruck, University of Innsbruck, 6020 Innsbruck, Austria; 4 Institute of Electron Microscopy and Nanoanalysis, University of Technology, 8010 Graz, Austria; peter.poelt@felmi-zfe.at; 5 Institute of Applied Physics, Vienna University of Technology, 1040 Vienna, Austria; gebeshuber@iap.tuwien.ac.at

**Keywords:** orientation-dependent reflection, structural color, butterflies, imprinting technique, instrument adaptation

## Abstract

The photonic structures of butterfly wing scales are widely known to cause angle-dependent colours by light interference with nanostructures present in the wing scales. Here, we quantify the relevance of the horizontal alignment of the butterfly wing scales on the wing. The orientation-dependent reflection was measured at four different azimuth angles, with a step size of 90°, for ten samples—two of different areas of the same species—of eight butterfly species of three subfamilies at constant angles of illumination and observation. For the observed species with varying optical structures, the wing typically exhibits higher orientation-dependent reflections than the individual scale. We find that the measured anisotropy is caused by the commonly observed grating structures that can be found on all butterfly wing scales, rather than the local photonic structures. Our results show that the technique employed here can be used to quickly evaluate the orientation-dependence of the reflection and hence provide important input for bio-inspired applications, e.g., to identify whether the respective structure is suitable as a template for nano-imprinting techniques.

## 1. Introduction

Structurally coloured butterflies have long been the subject of investigation by biologists and physicists alike [1,2,3,4,5,6,7] and have more recently inspired a range of bio-inspired applications. Structural colours arise from nanostructures found in wing scales that are covering the wing like shingles on a roof. Wing scales are made out of cuticle, a heterogenic mixture of chitin, protein and various lipids [7,8] and assembled in an enormous array of morphologies [1]. Commonly, wing scales are composed of a featureless lower lamina [9] and an elaborately structured upper lamina that can fold into morphologies ranging from arrays of folded ridges in *Morpho* (Fabricius, 1807) butterflies [10,11] to 3D photonic crystals of papilionid [12] and lycaenid [13] butterflies. These structures give rise to structural colours when incident light interferes with these morphologies as comprehensively described previously [10,14,15,16,17,18,19,20,21,22]. 

Multiple factors on different length scales affect how light interacts with the wings, which can include the scale morphology [23,24] and the scale stacking on the wing [25] on the macroscopic level, i.e., the scale lattice. On the microscopic level of a single wing scale, the pigmentation [12,25,26], thicknesses [27], and structural irregularities are the key parameters for light-matter interaction. For example, while being arranged regularly on the wing, the different heights of the ridge-lamellae of the upper scale lamina of morpho butterflies results in destructive interference for light of certain wavelengths while the ridge to ridge distance act as a (wavelength-dependent) diffraction grating [28].

Structural colours are well known for their pronounced iridescence and a range of methods have been used to investigate the origin of the structural and optical properties of butterfly wing scales. For example, light scattering measurements of isolated cover scales of *Morpho rhetenor* (Cramer, 1775) and *M. didius* (Hopffer, 1874) at normal incidence show that the reflected light is spatially confined into a diffraction band, perpendicular to the direction of the colour-causing diffraction structures, while a change of light incidence angle causes a pronounced blue-shift of the reflected hue [11,29]. Very early observations showed that the orientation-dependent reflection of different morpho species is based on the tilt angle of the scale lamina [5,30] and the orientation of the ridge-lamellae [2,3], though none of them provided quantification of this phenomenon or provided a broader survey into its origin. Recently, more advanced methods based on custom-built setups (imaging scatterometry or detection screen), have been presented [24,29].

Here, we introduce a facile method, based on off the shelf equipment, that allows evaluating the orientation-dependent reflection of butterfly wings as well as single scales. As a proof-of-concept, we investigate the reflection of ten samples from eight different butterfly species to determine the effect of (i) local structuring, (ii) pigmentation and (iii) disorder on the orientation-dependent reflection. This study provides a useful tool to estimate the visual effects of materials directly based on these bio-templates, before the actual replication takes place.

## 2. Materials and Methods

### 2.1. Butterfly Sample Selection

Different wing pieces and individual scales were measured from eight butterflies from three different subfamilies (details taxonomy see Table S1), including (i) four *Morphinae*: *Morpho helenor helenor* (Cramer, 1776; abbreviated as **Mh**), *M. helenor montezuma* (Guenée, 1859; **Mm**), *M. peleides* (Kollar, 1850; **Mp**) and *M. achilles* (Linnaeus, 1758; **Ma**), (ii) two *Satyrinae* butterflies *Caligo memnon peleus* (Stichel, 1904; **Cm**) and *Eryphanis polyxena* (Meerburgh, 1775; **Ep**) and (iii) two *Papilioninae*: *Papilio memnon* (Linnaeus, 1758; **Pm**), and *Parides sesostris* (Cramer, 1779; **Ps**). Butterflies were obtained commercially from a local butterfly farm (www.schmetterlingshaus.at, Friedmann, Vienna, Austria); except *Morpho helenor* which was provided by the Austrian Natural History Museum (Vienna, Austria).

### 2.2. Butterfly Sample Preparation

Wing pieces (2 × 2 cm) were cut from the wing with scissors and were subsequently fixed on a glass slide (Marienfeld, Lauda-Königshofen, Germany) by using adhesive tape (Scotch® Klebeband Magic™, 3M, Vienna, Austria). Single wing scales were isolated by gently pressing a standard cover slip (Assistent, Sonheim/Rhön, Germany) to a sectioned wing piece, so that single scales adhered to it by their upper lamina.

To allow single scale investigation within an inverted microscope, the cover slip was glued on to a glass slide using transparent nail polish (Colorama, DM, Vienna, Austria). A standard calibration procedure was applied including a reference measurement of the white standard (set to 100%) and a sample dependent background measurement, that is subtracted to achieve the final reflectance signal (Figures S1 and S2).

### 2.3. Imaging

Images of the isolated scales and wing pieces were recorded with a Leica DFC300FX (Wetzlar, Germany) digital camera connected to an inverted optical microscope Axiovert200 (Zeiss, Oberkochen, Germany) equipped with an Achroplan 20×/0.4 objective. The illumination was provided by a cold light source (KL 1500, LCD, 150 W, 15 V) via two-branch flexible light guides (Schott, Mainz, Deutschland). Both light guides were oriented at an angle of 40° with respect to the sample. In this setup, we experimentally determined that the signal was strongest at 40°. The light output over extended periods of time is stable (Figure S1). Images were processed using Photoshop CS3, Version 10 (Adobe Systems, San José, CA, USA). The same setup was used for the microspectrometry measurements detailed in the next section. Calibration and measurement (Table S2) were performed by using the software Image J 1.49d with an overlay of an imaged scale bar [31].

### 2.4. Microspectrometry

An Ocean Optics USB2000-Fl Spectrometer (Ocean Optics, Dunedin, New Zealand) was used for the spectral reflectance measurements. Light reflected from the samples, illuminated as described above, was confocally imaged onto a fibre (diameter 200 µm) and guided to the spectrometer. See Section 2.2 and Section 2.3 for complete information on the measurement system.

The actual measurement area on the sample has a diameter of 13 µm. This allows a measurement of the reflectance of the samples in the range of 400 nm up to 700 nm, though limitations of the available equipment. Calibration was performed with a white standard, made from barium sulphate 0.25 (±0.04) g (L. Kögl Pharma, Innsbruck, Austria). All samples were fixed on a slide and centered on the microscope stage before measuring. The orientation-dependent reflection measurement was performed by turning the slide in steps of 90°. The orientation-dependent reflection (*n* = 240) was measured for ten different upper wing pieces and their corresponding isolated upper scales; each sample was measured in the four above-mentioned orientations. Three scans per sample were processed (Table S2).

### 2.5. Ultrastructure

The samples were examined by scanning electron microscopy either with a JEOL JSM-IT300 (Pleasanton, CA, USA), a SEM Zeiss Gemini DSM 982 or a Zeiss Ultra 55 SEM (both: Zeiss, Oberkochen, Germany). The SEM samples were air dried and mounted with double-sided adhesive carbon tape on aluminum stubs. 

### 2.6. Statistics

Scale dimensions and orientation of features within the scale structures were measured using Image J 1.49d (Table S3). Spectral reflectance curves were smoothed using a “moving average” with a period of ~20 nm. Each graph represents the average of three scans and maxima were selected from the smoothed data. The difference value is the orientation-dependent variation calculated by the subtraction of the lowest value from the highest value of the orientation dependent peak reflectance values.

## 3. Results

**Figure 1:**
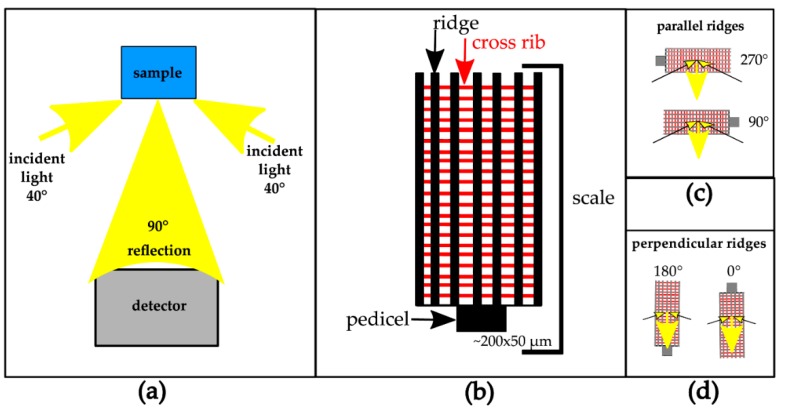
(**a**) Sketch of the experimental setup of the orientation-dependent reflectance measurements: the sample is illuminated with a two-branch flexible light guide at fixed angles of 40° and the reflection is detected along the surface normal of the sample (see Figure S3 for detailed photos of the experimental setup). (**b**) Sketch of the typical structure of the upper lamina of Lepidopteran wing scale, which features ridges (black lines) and cross-ribs (red lines). (**c**,**d**) Sketches of the sample orientation with ridges oriented either parallel (**c**, 90° and 270°) or perpendicular (**d**, 0° and 180°) to the incident light.

**Figure 2:**
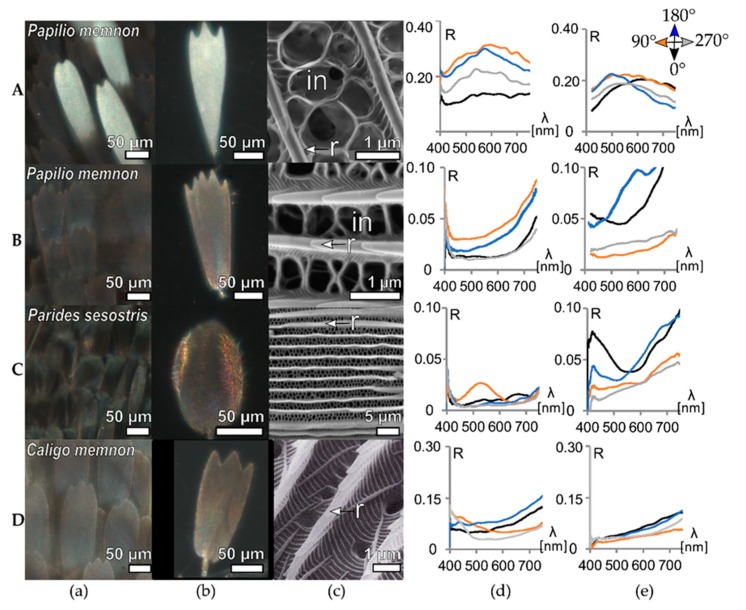
Pigmentary coloured butterflies with common grating structures. Row **A** *Papilio memnon* (white area), **B** *Papilio memnon* (black area), **C** *Parides sesostris* (black area), and **D** *Caligo memnon* (brown area). Column (**a**) shows optical micrographs (all oriented tip-up for convenience) of the scales arranged on the wing and column (**b**) shows optical micrographs of single wing scales. Column (**c**) shows SEM images of the scale ultrastructures. Column (**d**) displays orientation-dependent reflectance of the wing patches and (**e**) displays orientation-dependent reflectance of the single scales. In column c, *in* marks the irregular net of connecting cross-ribs and *r* marks the scale ridges. In column d, e, *R* represents reflectance and *λ* represents wavelength. The SEM image at panel Dc was reproduced with permission [1], Wiley, 1998.

**Figure 3:**
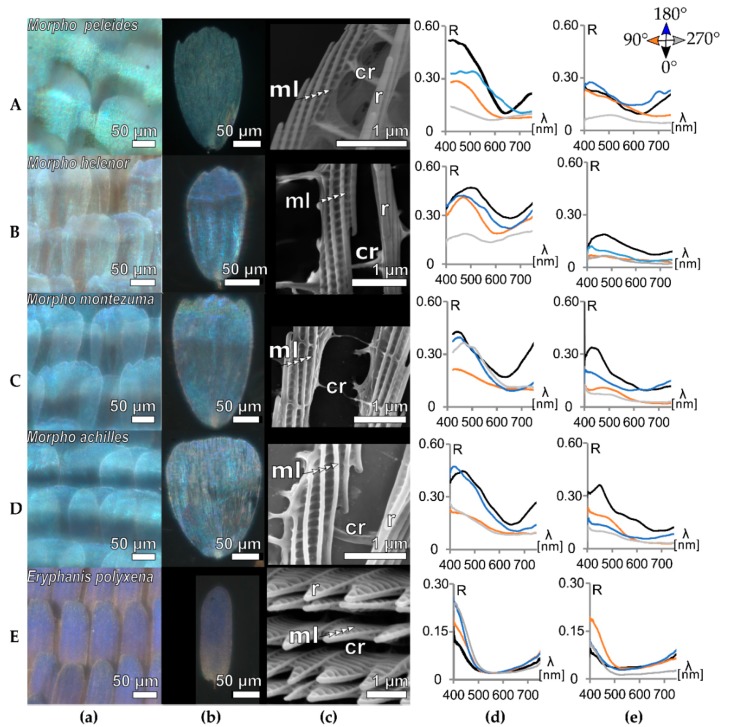
Multilayer reflectors of morpho butterflies and *Eryphanis polyxena*. (**A–E**), all with varying multilayer reflectors within their ridges. Column (**a**) shows optical micrographs (all oriented tip-up for convenience) of the scales on the wing, column (**b**) shows micrographs of the single wing scales. Column (**c**) shows SEM images of the scale ultrastructures. Column (**d**) displays the orientation-dependent reflectance of the wing patches and column (**e**) displays the orientation-dependent reflectance of the single scales. In column c, *ml* marks the multilayer structure in the scale ridges and *r* and *cr* mark the cross-ribs. In column d,e, *R* represents reflectance and *λ* represents wavelength.

**Figure 4:**
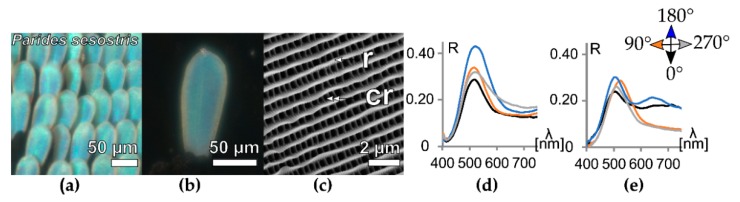
3D photonic crystal reflectors of *Parides sesostris*. Panel (**a**) shows an optical micrograph of the scales on the wing, panel (**b**) displays an optical micrograph of a single wing scale. Panel (**c**) shows an SEM image of the scale’s ultrastructure in top-view. The chart at position (**d**) displays the orientation-dependent reflectance of the wing patch and at panel (**e**) the chart represents the orientation-dependent reflectance of the single scales. The SEM image was reproduced here with permission [33], Royal Society Publishing, 2013. In panel (**c**), *r* marks the scale ridges and *Cr* marks the cross-ribs. In (**d**,**e**), *R* represents reflectance and *λ* represents wavelength.

The results of the angle-dependent reflection measurements (see Methods and Figure 1) are presented, in Figure 2, Figure 3 and Figure 4, for butterflies with pigmentary colours (Figure 2), ridge multilayers (Figure 3) and 3D photonic crystals (Figure 4), with increasing ultrastructural complexity. The rationale for performing these measurements is to quantify the relevance of the orientation-dependent reflection. The butterfly wing membrane is a rather featureless thin film [1,9,32] and therefore will likely not contribute to the orientation-dependent response. Orientation-dependent effects might be useful visual cues for mating, but also provide a source for bio-inspired applications, especially as biological structures can be directly used as templates. The applied spectrometry set up (for details, see Methods) is built around a standard light microscope, which allows easy adaptation of the setups, but also maintains the availability of normal microscopy. The light source was provided at an angle of ~40°, as this produced the best signal/noise ratio.

### 3.1. Simple Structured, Pigmented Scales

Pigments are likely the most prominent source of coloration across Lepidoptera [12,21,32,34], where pigment is diffusely arranged within the wing scales. Here we investigated the scales of two different papilionid (*Papilio memnon* and *Parides sesostris*) butterflies and one nymphalid (*Caligo memnon*) butterfly which also has pigmented wing scales (Figure 2). The selected wing piece of *Papilio memnon* has predominantly white scales surrounded by a black margin (Figure 2A,B), while the investigated areas of *P. sesostris* and *C. memnon* are black and brown, respectively (Figure 2C,D). Figure 2c shows the ultrastructures of the pigmented wing scales. All papilionid butterflies (Figure 2A–C) share the common basic *Bauplan* (sensu [27]). The scales feature an elaborate, irregular net of cross-ribs between ridges (r). The mean ridge-to-ridge distance is ~2 µm for *P. memnon* and ~1.5 µm for *P. sesostris* (for more detailed information on the ultrastructure, see Table S3). *Caligo memnon* (Figure 2D) has brown pigmented upper scales. These scales do not feature a net of cross-ribs but rather a connected layer of many ribs with a few open spots and a mean ridge-to-ridge distance ~1.7 µm.

We measured the orientation-dependent reflectance of these samples for wing pieces (Figure 2d) and single wing scales (Figure 2e). The white wing piece of *Papilio memnon* shows orientation-dependent reflectance ranging from 0.14 to 0.31 (Figure 2A(d)). The reflectance range is much lower for the single wing scale: 0.19 to 0.23. The black wing piece and the black scales of the examined *Papilionidae* butterflies show a low reflectivity due to pigmentation. Both the black *Papilio memnon* area (Figure 2B(d,e)) and the black area of *Parides sesostris* (Figure 2C(d,e)) show a strong orientation-dependence with a two-fold increase in reflection (Table S2). For the investigated *Papilio memnon* area (Figure 2B(d)), reflectance ranges from 0.04 to 0.09, while for the *Parides sesostris* area (Figure 2C(d)) reflectance ranges from 0.02 to 0.03. For individual scales (Figure 2B(e),C(e)), the orientation-dependent reflection is more pronounced than those of the wing patches reaching as much as a four-fold increase. The increase in reflectance values from wing to scale may derive from scale stacking on the wing, as this could weaken/average the effects of single scales. The results in Figure 2C(d) (90° orientation) and Figure 2B(e) (180° orientation) clearly demonstrate the impact of the scale stacking angle and the scale curvature on the measured orientation-dependent reflection.

### 3.2. Ridge Multilayers

Many colours of Lepidoptera are not pigmentary, but based on interference with elaborate nanostructures in the wing scales. These wing scales feature more elaborate structures than the pigmentary scales of Figure 2. To investigate the effect of these nanostructures on the orientation-dependent reflectance, we examined the ridge-multilayer reflectors of five taxa of nymphalid butterflies (for taxon list see Tables S1 and S2). Figure 3 presents the wing scale lattice, ultrastructure and orientation-dependent reflectance of the investigated samples. All morpho samples share shimmering blue rows of cover scales with brown ground scales below as seen in the high magnification images (Figure 3a). Isolated morpho cover scales are bright blue, as seen in Figure 3b. The scale structure (Figure 3c) highlights the well-documented ultrastructure composed of ridge lamellae folded into a multilayer. This ultrastructure is typical to morpho [11,27,35] butterflies and can be found in all samples. The measured ridge-to-ridge distances of the morpho samples are 0.9–1.5 µm and these can act as diffraction grating [35] (Table S3). The ridge-lamellae count varies between samples (from 3 to 6 layers) with slight variation in their tilt with respect to the scale base (9° ± 2° up to 14° ± 2°, see Table S3 for more details). The cross-ribs in these wing scales are minor in appearance. *E. polyxena* (Figure 3E) is different to the morpho butterflies of Figure 3A–D in that the layers in the ridges have a very steep tilt angle (see Figure S4). This structural difference leads to a variation in the direction-dependence of the resulting iridescence but not in the orientation-dependent reflection.

The orientation-dependent reflection (Table S2) of the measured morpho wing pieces shows a pronounced reflectance band in the blue wavelength range and the spectra vary by a factor of two (from 0.14–0.22 up to 0.43–0.52), while the single scale shows larger orientation-dependence by a factor of up to three (from 0.09–0.1 vs. 0.19–0.36). The orientation-dependent reflection of *Eryphanis polyxena* also shows an intensity variation of approximately a factor of two (wing piece 0.11–0.22, single scale 0.08–0.17), though these scales likely feature pigment in addition to the structure. We note that the different scale structures could cause a different orientation-dependent optical effect, however, we do not observe it. This leads us to hypothesize that the common grating structure, composed of ridges and cross-ribs, rather than the local ultrastructure, determines the angle-dependent optical effect.

### 3.3. Photonic Crystals

One of the most complexly ordered photonic structures in nature are photonic crystals. To investigate the effect of this complex nanostructure, we investigated the deep green wing patch of the Emerald-Green Cattleheart butterfly, *Parides sesostris* [33,36,37,38] (Figure 4). The cover scales in the green wing patch feature a gyroid photonic crystal that is covered by a thick, pigment-filled layer of longitudinally ordered ridges of fluted micro-ribs and cross-ribs (Figure 4c) [33]. The ridge separation of this layer in top-view is 0.5 ± 0.06 µm and features densely ordered cross-ribs with a mean distance of 0.4 ± 0.03 µm (Table S3).

The angle-dependence of single wing scales has been investigated before [38], but the orientation-dependence has not been quantified. The reflectance of the green *Parides sesostris* wing piece shows a pronounced band in the green wavelength range with an orientation-dependent variation of ~1.5 (0.29 up to 0.43). Single scales show a similar amount of variance (0.24 up to 0.30).

### 3.4. Comparative Analysis of the Orientation-Dependent Reflectance

**Figure 5:**
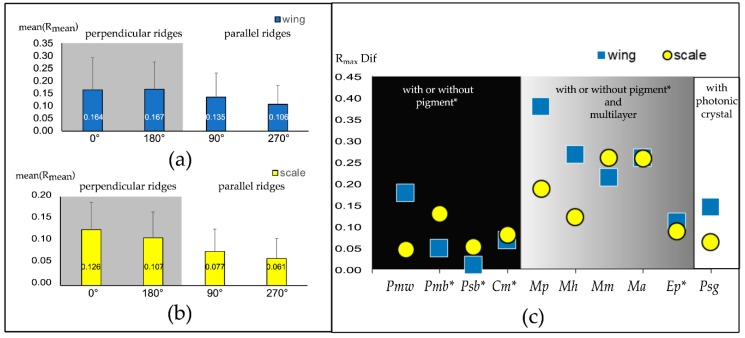
Mean orientation-dependent reflectance for all investigated butterfly species. The chart representing wing patches is in panel (**a**) and the chart representing single scales is in panel (**b**). The reflectance perpendicular to the ridges (0° and 180°) is higher than the reflectance parallel to the ridges (90° and 270°). Panel (**c**) displays the difference in orientation-dependent reflectance for the wing patches (blue squares) and single scales (yellow circles) ordered by increasing structural complexity of the scale ultrastructure and the dominant colouration mechanism. (species abbreviations see Section 2.1).

The ten butterfly areas investigated span a large variety of structural modifications observed in Lepidoptera, ranging from the most abundant commonly occurring basic scales with or without pigments (Figure 2), to the more complex ridge-lamella multilayer (Figure 3), up to highly complex 3D photonic crystals (Figure 4). All samples (wing pieces and corresponding single scales) show an orientation-dependent reflectance. Figure 5 displays plots comparing the mean orientation-dependent reflectance averaged over all wing pieces (Figure 5a) and all single scales (Figure 5b). Figure 5c compares the orientation-dependent reflectance for the different scale morphologies.

Figure 5a,b shows that the orientation-dependent reflectance is strongest in the direction when the incident light is perpendicular to the ridges. During the measurements, the sample is oriented with the scale tip either perpendicular (0° and 180°) or parallel (90° and 270°) to the plane of incident light. The reflectance of the wing areas with scale ridges oriented perpendicular (0° and 180°) is roughly 40% higher than when the scale ridges are parallel (90° and 270°). On the single-scale level (Figure 5b), the reflectance is about 70% higher in directions perpendicular to the ridges.

To understand if this effect is stronger for different ultrastructures, we plotted the maximum reflectance contrast (highest R − lower R)—based on the peak values per orientation—in Figure 5c for wing patches (blue squares) and wing scales (yellow circles). As shown in Figure 2, Figure 3 and Figure 4, the orientation-dependent effect is observed in all samples, but seems to be stronger for scales with more pronounced ridges (Figure 5c). While the orientation-dependent reflectance is ~0.1 for pigmented scales (black background), it can reach values of ~0.2 for the ridge-multilayer structures of Figure 3 (gray). The heavily pigmented black scales of *P. sesostris* show similarities to the green-coloured structured wing scales of Figure 4 and to the pigmented white scales of *Papilio memnon*. The differences between the wing and individual scale measurements are mainly due to scale stacking and the angle of the scale relative to the wing base. Figure S5 shows further details about the individual ordinal ranking of the orientation-dependent maximum reflection values.

## 4. Discussion

Here, we have investigated the orientation-dependent reflection in 90° (azimuth) steps by using a new, simple, and fast optical characterisation method and compared the results from eight different butterfly taxa. Our selection of butterflies from different families ensured a variety of structural architectures to aid in the goal of measuring their influence on the orientation-dependent reflection. The results show that rather than the local higher-order structuring of the scale, the hierarchical lattice of ridges and cross-ribs, quintessential to the morphology of any Lepidopteran wing scale [1], is the key parameter that influences the measured angle-dependence. Simply speaking, the orientation-dependent reflection originates from light interacting with the anisotropic grating formed by the ridges and cross ribs of the scale. The data presented here confirm the hypothesis that the precise alignment of structurally coloured butterfly scales (or to be more precise, their wing scale lattice) has to be considered when interpreting optical measurements of butterfly samples and their replicas.

### 4.1. Orientation-Dependent Reflection

Our results show that the sample orientation has an important impact on the reflection. The results of Figure 2, Figure 3 and Figure 4 confirm a previous hypothesis that this is caused by the common lattice structure of wing scales (see page 87 of ref. [39]). 

The common scale grating composed of ridges and cross-ribs is the key parameter that causes the measured orientation-dependent reflectance and not the colour reflecting photonic structures that might be found additionally. The results of Figure 3 show that structurally thicker ridges, like those of morpho butterflies, provide a larger optical effect. Single scales consistently showed a larger contrast, which could be due to the more consistent scale orientation as it eliminates tilting artefacts caused by the way scales are mounted on the wing. Future investigations examining arrangement effects, e.g., quantification of the impact of the scale stacking angle, need to be performed in more detail. 

The impact of scale ultrastructures of the investigated butterflies are well known [1,25,29,30,37,38,40,41]. For example, the black scales of samples of the papilionid taxa are known to enhance the appearance of black [42]. Though being a subtle difference, the present study adds that the enhancement of the blackness is in fact orientation-dependent. The arrangement of the lattice structure (ridges and cross ribs) of the scale is crucial for the orientation-dependent reflection. Until now, this fact was only confirmed to be true for the morpho butterflies [35,39]. The present study indicates light reflected perpendicular to the ridges has a higher intensity than that reflected in parallel directions (Figure 5, see also [10]).

### 4.2. Measurement Method

Previous studies have already measured the angle-dependent reflectance for different geometries, including aligned wing patches, and single scales measured from abwing and adwing sides [9]. The results of the applied, fast, and facile technique are indeed in good agreement with the results of the known abwing and adwing measurements defining top side and bottom side of single wing scales [27,43].

The present work adds another tool to the growing toolbox of physical approaches [44] to measure the light scattered by wing scales (Figure 1). With this measurement arrangement, the reflection of the sample can be measured at arbitrary horizontal angles. The sample can be characterized a larger variety of ways than those shown here, as this will allow control over the illumination angle between the acceptance angle of the objective and near grazing incidence or a change in the illumination conditions. Another advantage is that this setup can be universally applied to a common light microscope with off-the-shelf components and does not need custom-built parts [24]. Naturally, the presented technique shares the drawbacks of common light microscopes, e.g., a limited spectral range in the UV.

### 4.3. Bio-Inspired Applications

The presented technique and the structural and spectral results are useful for biomimetic principle transfer, because the basic knowledge of structural colour properties serves replication techniques in their choice of templates [17,45]. The examined reflection in the visible range of light shows the structure-specific potential for technical applications visible to the human eye [45,46]. The orientation-dependent tunability of the reflection by grating indicates hue or intensity dependent alignments which can be used as an accessory tool of precision equipment like cameras or especially within a compass [46] in the case of magnetism failure. Finally, the structural replication of those properties allows for the usage of sustainable and degradable materials, thus conferring a benefit for the technique and society.

## Supplementary Materials

The following are available online at https://www.mdpi.com/2313-7673/5/1/5/s1, Figure S1: Stability of the lamp, Figure S2: Measured signal noise, Figure S3: Experimental set up, Figure S4: Scanning electron images of a *Morpho* cover scale structure and of a *Eryphanis polyxena* scale, Figure S5: Ordinal rankings of the orientation-dependent peak reflectance, Table S1: Taxonomic breakdown of the investigated butterflies, Table S2: Maximal orientation-dependent reflectance of ten wing pieces and single scales, Table S3: Dimensions of the scale features.
