# Supplementary material for: Orientation-Dependent Reflection of Structurally Coloured Butterflies
Source: Biomimetics (Basel). 2020 Feb 3;5(1):5. doi: 10.3390/biomimetics5010005 (PMC7148507; doi:10.3390/biomimetics5010005)
Supplement: Supplementary file 1 [file biomimetics-05-00005-s001.pdf]

Supplementary Information

for

Orientation-dependent reflection of structurally coloured butterflies

by

Sigrid Zobl, Bodo D. Wilts, Willi Salvenmoser, Peter Pölt, Ille C. Gebeshuber, and Thorsten Schwerte

<u>Contents</u>

Tables S1–S3

Figures S1–S5

Table S1 Taxonomic breakdown of the investigated butterflies into eight taxa representing two families, three subfamilies, four tribes, five genera, and seven species. One species is represented by two subspecies (*Morpho helenor helenor* and *Morpho helenor montezuma*).

Order	Superfamily	Family	Subfamily	Tribe	Species	Subspecies
Lepidoptera	Papilionoidea					
		Nymphalidae	Morphinae	Morphini	*Morpho helenor*	*M. h. helenor*<br>*M. h. montezuma*
					M. peleides	
					M. achilles	
		Papilionidae	Satyrinae	Brassolini	*Caligo memnon*	*Caligo memnon peleus*
					Eryphanis polyxena	
			Papilioninae	Troidini	*Parides sesostris*	
				Papilionini	*Papilio memnon*	

Table S2. Maximal orientation-dependent reflectance of ten wing pieces and single scales ordinally ranked. The difference value (DIF) is the maximum deviation of the peak reflectance (max) of each sample (see Fig. 1). Species abbreviations given in the main text, *w* abbreviated white, *b* black and *g* green areas respectively. O represents the orientation O, R the peak reflectance; coloured fields refer to the spectral ranges shown in the legend above the table.

**Colour** [nm]	435 –480	490 –500	560 –580	595 –605
400 – 435	480 –490	500 –560	580 –595	605 –750

**SAMPLE**	**wing**				**scale**			
	**O [°]**	**max [nm]**	**R**	**DIF**	**O [°]**	**max [nm]**	**R**	**DIF**
Pmw	90	584	0.31		0	740	0.23	
	180	564	0.30		180	489	0.23	
	270	530	0.23		270	526	0.19	
	0	557	0.14	0.18	90	494	0.19	0.05
Pmb	90	740	0.09		0	740	0.17	
	180	739	0.08		180	740	0.14	
	0	739	0.05		270	740	0.04	
	270	739	0.04	0.05	90	740	0.04	0.13
Psb	90	518	0.03		0	739	0.10	
	180	740	0.02		180	740	0.09	
	0	738	0.02		90	730	0.06	
	270	738	0.02	0.01	270	731	0.05	0.05
Cm	180	740	0.16		0	749	0.14	
	0	737	0.12		180	739	0.11	
	90	427	0.10		270	739	0.09	
	270	411	0.09	0.07	90	735	0.06	0.08
Mp	0	415	0.52		180	414	0.28	
	180	451	0.34		0	411	0.23	
	90	433	0.29		90	411	0.22	
	270	411	0.14	0.38	270	490	0.09	0.19
Mh	0	489	0.47		0	459	0.19	
	180	459	0.42		180	412	0.11	
	90	460	0.41		90	413	0.07	
	270	740	0.2	0.27	270	460	0.07	0.12
Mm	0	427	0.43		0	417	0.34	
	180	469	0.42		180	413	0.20	
	270	456	0.36		90	461	0.11	
	90	421	0.22	0.21	270	412	0.08	0.26
Ma	180	411	0.47		0	439	0.36	
	0	439	0.45		90	411	0.20	
	270	413	0.23		180	413	0.14	
	90	413	0.21	0.26	270	413	0.1	0.26
Ep	270	411	0.22		90	411	0.17	
	180	411	0.20		180	739	0.09	
	90	411	0.15		270	411	0.09	
	0	412	0.11	0.11	0	739	0.08	0.09
Psg	180	509	0.43		180	494	0.30	
	0	505	0.34		90	513	0.29	
	90	509	0.32		270	498	0.26	
	270	507	0.29	0.14	0	495	0.24	0.06

Table S3. Dimensions of the scale features based on the scanning electron microscopic images. The table gives the sample size (n), the tilt angle (TA) of the multilayer in the ridge lamellae with respect to the lower scale lamina (Figure S4 gives a detailed view of the parameters), the ridge to ridge distance, the ridge width and the cross-rib to cross-rib distance. Abbreviations as introduced in the main text.

common scale structures [μm]									
sample	**counts of layers (multilayer)**	**TA [°]**	**n**	**ridge to ridge**	**n**	**ridge width**	**n**	**Cross-rib to cross-rib**	**N**
Pmw				1.9±0.2	10	0.31±0.02	5		
Pmb				2±0.2	10	0.36±0.05	10		
Psb				1.5±0.1	10	0.3±0.05	8		
Cm[+]				1.5–1.9	2			011±0.02	9
Mp	3–4	9±2	5	0.92±0.1	5			1.5±0.4	5
Mh	4–5	12±3	5	1±0.09	10			3±0.5	5
Mm	4–5	10±2	8	1±0.2	5			3±1	5
Ma	5–6	14±2	5	1.5±0.1	10	0.3±0.06	5	3±1	5
Ep	>12	0		0.5±0.1	5	0.13±0.02	10		
Psg[++]				0.5±0.06	5			0.4±0.03	10

[+]*Ghiradella, H. Hairs, Bristles, and Scale figure 36 in Microscopic Anatomy of Invertebrates: (eds Harrison, W.&Locke, M.) Fig. 36 (Wiley, 1998).*
[++]*Yoshioka, S., Fujita, H., Kinoshita, S. & Matsuhana, B., Figure 1 e, Alignment of crystal orientations of the multi-domain photonic crystals in Parides sesostris wing scales. Journal of The Royal Society Interface* ***11****, 2013102, Fig. 1e; 10.1098/rsif.2013.1029 (2013).*

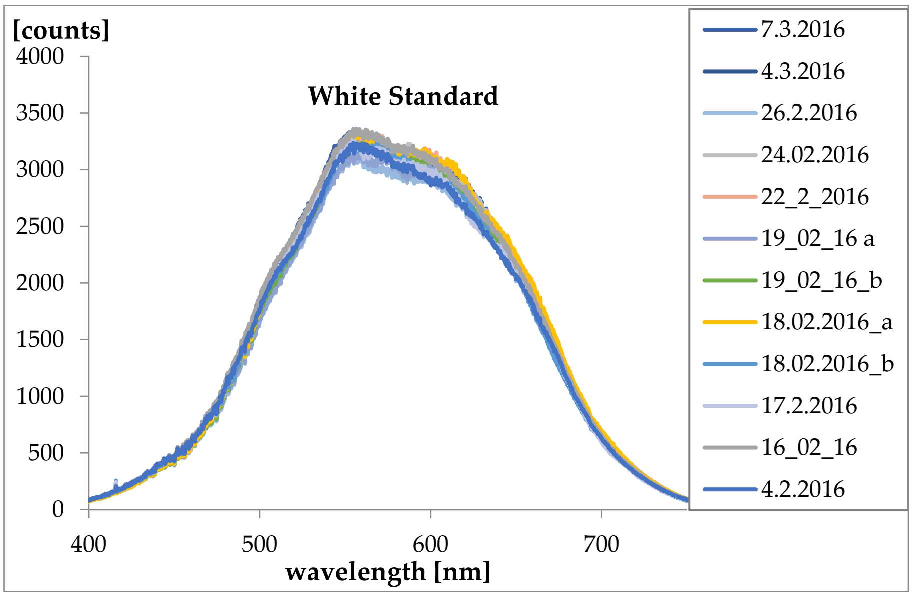

Figure S1. Stability of the lamp measured on a white standard over the period of one month.

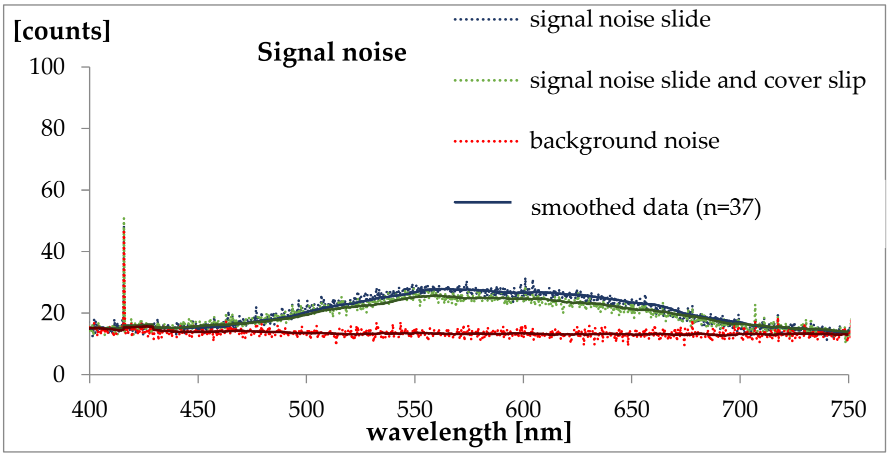

Figure S2. Measured signal noises with or without the slide, with or without the coverslip show the sample dependent varying background subtraction.

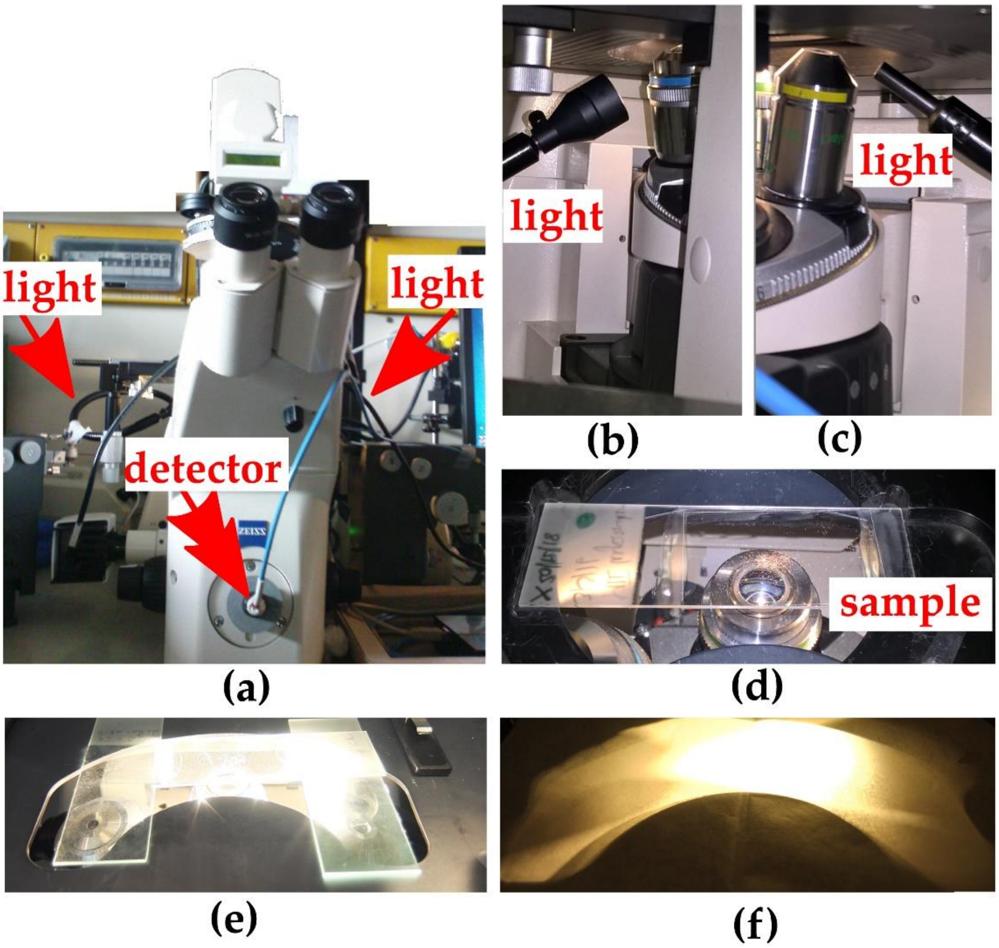

Figure S3. Experimental set up. (a) A common inverse light microscope with the detector (double arrows) and the two branch flexible light guides (single arrow). (b,c) detailed view of the illumination from sides. (d) On view onto the sample stage. (e,f) illumination profiles on a scattering paper show that the illumination is homogenous over an extended area.

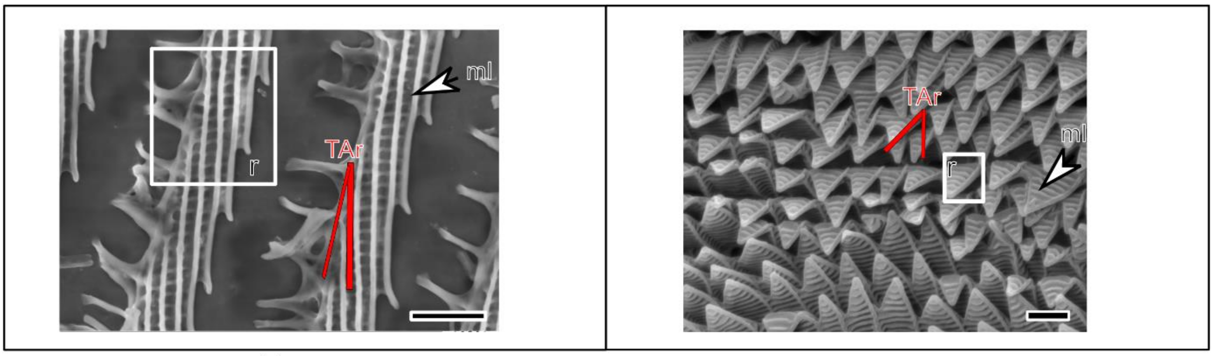

(a) (b)

Figure S4. Scanning electron images of a *Morpho* cover scale structure (**a**) and of a *Eryphanis polyxena* scale (**b**). Shown are the ridges (r), and the definition of the tilt angle of the ridges in the multilayer (ml). Scale bars 1 μm.

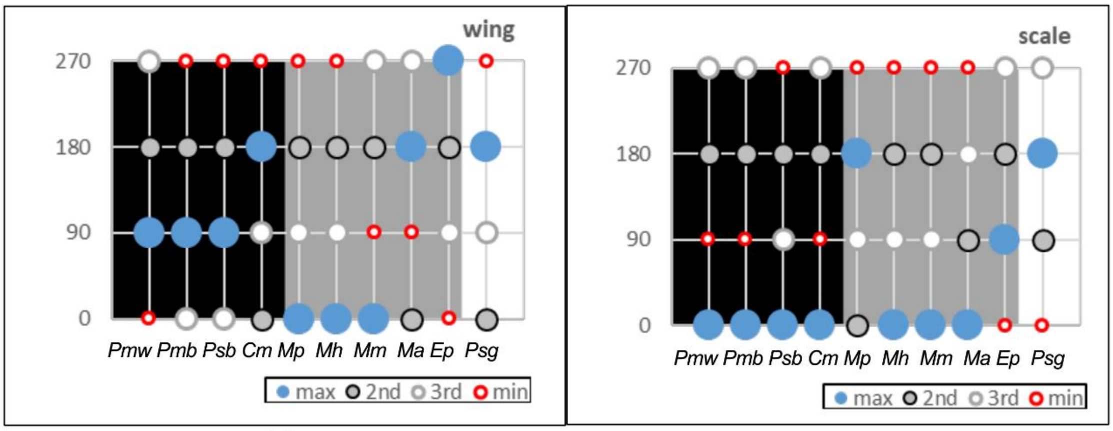

(a) (b)

Figure S5. Ordinal rankings of the orientation-dependent peak reflectance (**a**) of the wing pieces and (**b**) single wing scales. The different sizes and colours give a ranking of the peak reflectance values. The black, grey and white background accord to increasing complexity of the wing ultrastructure and the dominant colouration mechanism. The species abbreviations are defined in the main text.
